# All-trans retinoic acid alleviates collagen-induced arthritis and promotes intestinal homeostasis

**DOI:** 10.1038/s41598-024-52322-x

**Published:** 2024-01-20

**Authors:** Yiqi Zhang, Yating Luo, Jiangchun Shi, Yumeng Xie, Huangfang Shao, Yun Li

**Affiliations:** 1 Department of Nutrition and Food Hygiene, West China School of Public Health and West China Fourth Hospital, Sichuan University, Chengdu, Sichuan China; 2 Healthy Food Evaluation Research Center, Sichuan University, Chengdu, China

**Keywords:** Chronic inflammation, Rheumatoid arthritis

## Abstract

All-trans retinoic acid (ATRA) has emerged as a promising adjunctive treatment for rheumatoid arthritis. However, the mechanism by which ATRA mitigates arthritis remains unclear. In this study, we aimed to explore ATRA alleviation of arthritis and the role of ATRA in regulating intestinal homeostasis. Thus, we established a collagen-induced arthritis (CIA) model in Wistar rats. After 6 weeks of ATRA treatment, the arthritis index of CIA rats decreased, synovial inflammation was alleviated, and the disruption of Th17/Treg differentiation in peripheral blood was reversed. Additionally, the Th17/Treg ratio in the mesenteric lymph nodes decreased and the expression of Foxp3 mRNA increased and that of IL-17 mRNA decreased in the colon and ileum. Microscopically, we observed reduced intestinal inflammation. Transmission electron microscopy revealed that ATRA could repair tight junctions, which was accompanied by an increase in the expression of Claudin-1, Occludin and ZO-1. Moreover, ATRA regulated the composition of the gut microbiota, as was characterized based on the reduced abundance of Desulfobacterota and the increased abundance of Lactobacillus. In conclusion, ATRA demonstrates the potential to alleviate arthritis in CIA rats, which might be correlated with modulating the gut microbiota and regulating the intestinal immune response. Our findings provide novel insights into ATRA-mediated alleviation of arthritis.

## Introduction

Rheumatoid arthritis (RA) is a chronic autoimmune disease that is characterized by synovial proliferation, bone destruction, neovascularization and inflammatory cell infiltration, which ultimately leads to joint and skeletal damage^1,2^. As a result, RA is associated with a lower quality of life, partial or total work incapacity and an increased risk of developing other comorbidities^3^. According to the 2017 Global Burden of Disease data, between 1990 and 2017, RA increased 7.4% in age-standardized prevalence, RA incidence increased 8.2%, and the growth in disability-adjusted life years (DALYs) increased from 0.24 to 0.31% of total years^4^. The significant impact of RA on families and society underscores its status as a pressing global public health concern. However, clinical treatment outcomes for many RA patients are suboptimal, and the potential toxic side effects of medications cannot be ignored^5,6^. Thus, the exploration of new approaches for managing RA is important.

Changes in the microbial composition of the lungs, oral cavity and gut in both preclinical and established RA individuals imply the potential involvement of mucosal dysbiosis in RA pathogenesis^7–10^. The intestine is populated by the largest number of innate and adaptive immune cells in the body and is therefore often considered to be the largest immune organ^11^. The gut microbiota may influence intestinal inflammation through T-cell regulation. For instance, the colonization of germ-free mice with *Bacteroides fragilis* expands regulatory T cells (Tregs), thereby promoting the production of anti-inflammatory cytokines in the intestine^12^. Some research suggests that in patients with compromised intestinal mucosal barriers, immune cells may enter the joints from the intestines via the bloodstream^13–15^. The gut-joint axis may play an important role in the onset of RA.

As early as the 1980s, Mezes et al.^16,17^ found that the serum retinol content of RA patients was significantly lower than that of healthy people. Subsequently, several studies found that the intake of vitamin A by RA patients was significantly lower than that by healthy people^18–20^, which made individuals speculate that the onset of RA may be related to vitamin A deficiency. All-trans retinoic acid (ATRA) is a key vitamin A derivative, and several animal experiments have demonstrated that it has anti-arthritis potential based on inhibiting the production of proinflammatory cytokines and mitigating RA-related symptoms^21–23^. Furthermore, previous research has highlighted the role of retinoic acid in maintaining mucosal homeostasis in the intestine. ATRA regulates immune cells, the gut microbiota, and the intestinal mucosal barrier; moreover, these components interdependently influence each other^24,25^. Vitamin A deficiency can lead to dysbiosis in the gut microbiota, as observed in animal experiments, resulting in increased intestinal epithelial permeability and increased proinflammatory cytokine secretion^25^. Additionally, emerging research has highlighted the role of vitamin A and its metabolites in modulating intestinal permeability by regulating tight junction (TJ) protein expression^26,27^.

As a potential supplementary therapeutic agent for RA, the mechanism by which ATRA alleviates arthritis remains unclear. Previous studies have mostly focused on the effects of ATRA on T-cell differentiation, inflammatory factors, and osteoclast formation. To the best of our knowledge, there is currently no research exploring the mechanism by which ATRA alleviates arthritis from the perspective of the gut-joint axis. In this study, we aimed to establish a collagen-induced arthritis (CIA) model and explore the potential mechanisms by which ATRA alleviates RA by exploring its effects on the gut microbiota, the intestinal epithelial barrier and intestinal immunity.

## Materials and methods

### Animals and treatment

A total of 35 healthy female specific pathogen-free (SPF) Wistar rats aged 6–8 weeks and weighing 200 ± 20 g were purchased from Vital River Laboratories (Beijing, China). All animal experiments were reviewed and approved by the Ethics Committee of Sichuan University West China Medical for Animal Experimentation (K2019002-2). All experiments were conducted in accordance with the Guide for the Care and Use of Laboratory Animals and complied with the requirements of the National Act on the Use of Experimental Animals (China). The authors followed the ARRIVE guidelines to minimize animal suffering.

After 1 week of adaptive feeding, 10 rats were selected for the blank group according to the random number table method, and the remaining 25 rats were intradermally immunized with 200 μg of cattle type II collagen (Chondrex, Redmond, WA, USA) emulsified in incomplete Freund's adjuvant (Chondrex) at the base of the tail and then given a booster immunization 7 days later to establish the CIA model. The blank group of rats were subcutaneously injected with the same volume of sterile saline.

**Figure 1:**
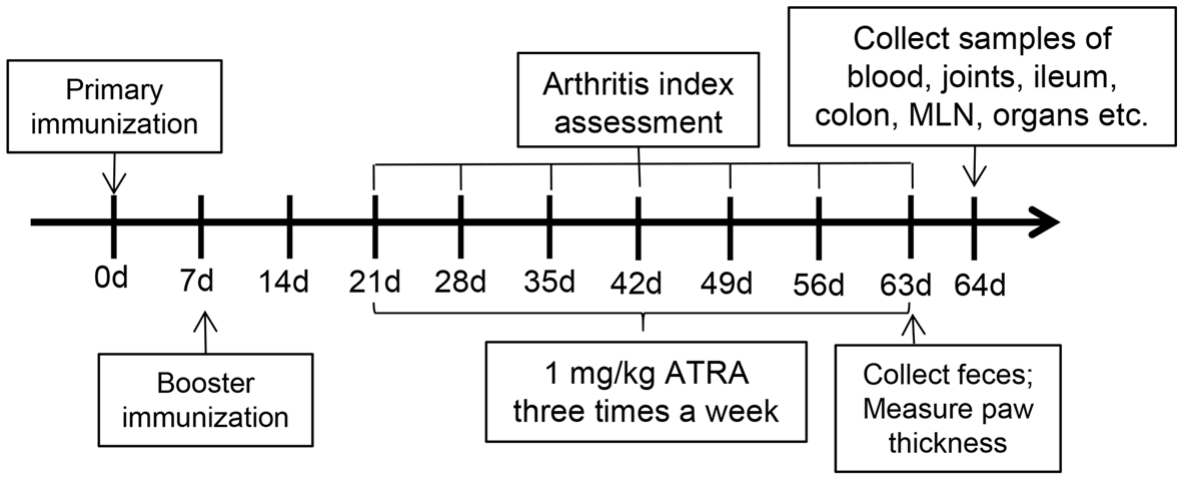
Experimental study design.

Finally, the model was successfully established in 22 rats, and these rats were randomly divided into the control group and the ATRA group. We utilized the following three groups: the blank group (healthy Wistar rats gavaged with corn oil three times a week), the control group (CIA model Wistar rats gavaged with corn oil three times a week) and the ATRA group(CIA model Wistar rats gavaged with 1 mg/kg ATRA (Sigma‒Aldrich, St. Louis, MO, USA) dissolved in corn oil three times a week). The body weight and the arthritis index (AI)^28,29^ were measured and recorded every week. The thickness of the paw was measured using callipers 9 weeks after the primary immunization. The rats were sacrificed with anaesthesia using ketamine/xylazine (100/10 mg/kg) after 6 weeks of treatment. The entire process is shown in Fig. 1.

### Histopathology and immunohistochemistry (IHC)

Ankle joints, livers, kidneys, ileums, colons, etc., were fixed with formalin and embedded in paraffin, and ankle joints were further decalcified with 10% ethylenediaminetetraacetic acid (EDTA) solution for 8 weeks. Then, the paraffin-embedded specimens were cut into serial sections (4 μm thick) and stained with haematoxylin and eosin (H&E). Images of three fields of view were randomly captured using a microscope (Leica, Wetzlar, Germany).

Slides of ankle joints, ileums and colons were scored blindly by 2 individuals. The following scoring system was used to assess the ankle joints: 0 = normal joints, absence of inflammation; 1 = evidence of soft tissue inflammation, synovial hyperplasia and/or cell infiltration into the synovial space (pannus); 2 = Grade 1 and definite erosion of articular cartilage; 3 = Grade 2 and definite erosion of articular bone, but joint architecture mostly intact; and 4 = Grade 3 with bone erosion resulting in a major loss of joint architecture^30^. The histological score for ileums and colons were determined based on a scale that graded the extent of inflammatory infiltrate (0–5), crypt damage (0–4), ulceration (0–3), and the presence or absence of edema (0 or 1)^31^.

Sections were then deparaffinized, rehydrated through graded ethanol solutions, and subjected to endogenous peroxidase activity blocking. Claudin (1:250 dilution, Bioss, Beijing, China), Occludin (1:600 dilution, Servicebio, Wuhan, Hubei, China), and ZO-1 primary antibodies (1:250 dilution, Bioss, Beijing, China) were applied in a humidified chamber at room temperature overnight. Afterwards, the sections were incubated with secondary antibody (1:500 dilution, Biosharp, Hefei, Anhui, China) at room temperature for 30 min. Subsequently, sections were processed with SABC, incubated at room temperature for 30 min, treated with DAB colour solution, and rinsed with water. Haematoxylin staining was performed for 2 min, followed by dehydration in graded alcohol solutions, and slides were sealed with neutral gum. Immunostained sections were examined and imaged under a light microscope at 200 × magnification, and ImageJ software (http://rsb.Info.nih.gov/ij) was applied for quantitative measurement by randomly selecting three fields in every slide. The mean optical density value was calculated by dividing the cumulative optical density by the total area.

### Western blotting analysis

To extract cellular and tissue proteins, RIPA buffer supplemented with protease inhibitor cocktail (Beyotime, Shanghai, China) was used to lyse samples at 4 °C for 30 min. Equal amounts of protein were loaded onto SDS‒PAGE gels and then transferred onto polyvinylidene difluoride (PVDF) membranes (Millipore, Billerica, MA, USA). After blocking the membranes with 5% non-fat milk at room temperature for 1 h, primary antibodies were added and the samples were incubated overnight at 4 °C; then, the samples were incubation with peroxidase-conjugated secondary antibodies at room temperature for 1 h. Immunoreactive proteins were analysed using an enhanced chemiluminescence kit (Millipore). Quantitation of the relative intensity of western blotting bands was performed using ImageJ software. Western blotting analysis were performed in technical triplicates.

### Enzyme-linked immunosorbent assay (ELISA)

Whole blood was collected from the rats and left at room temperature for 2 h. After coagulation, the blood was centrifuged at 3000 rpm for 10 min to obtain serum, which was then stored at − 80 °C until further use. The dissected ileal and colon tissues were washed with saline, dried using filter paper, and weighed. These intestinal tissues were then cut into pieces to create a 10.00% tissue homogenate (intestinal tissue (g): cell lysate (mL) = 1:9) with cell lysate (Boster, Wuhan, China) in an ice bath. After centrifugation at 8000 rpm for 10 min at 4 °C, the supernatant was collected and stored at − 80 °C for future use. The concentrations of tumour necrosis factor-α (TNF-α), interleukin (IL)-6, IL-17, IL-10, secretory immunoglobulin A (SIgA), vascular endothelial growth factor (VEGF) and vascular endothelial growth factor receptor 2 (VEGFR2) were determined using ELISA kits (4A Biotech, Beijing, China). The absorbance at 450 nm was immediately measured in each well using a Multiskan Spectrum (Bio-Rad, California, USA). Sample concentrations were calculated based on the standard curve. ELISA was performed on each sample in triplicate.

### Transmission electron microscopy (TEM) analysis

Five-millimetre segments of fresh ileum and colon tissues were flushed with PBS and fixed in 3% glutaraldehyde at 4 °C for 4 h. After rinsing in PBS, the tissue underwent additional fixation in PBS containing 1% osmium tetroxide (Seebio, Shanghai, China) for 2 h at room temperature, followed by further PBS rinsing and dehydration. Next, the tissues were embedded in Epon 812 (SPI, West Chester, PA, USA) and left to cure in an oven at 60 °C for 48 h. Sections with a thickness of 80 nm were cut using a diamond knife on an ultramicrotome (EM UC7, Leica). These sections were placed on single-hole grids coated with Formvar and carbon and then double-stained in aqueous solutions of 8% uranyl acetate for 25 min at 60 °C and lead citrate for 3 min at room temperature. Images of three fields of view were randomly captured using a JEM-1400 FLASH Transmission Electron Microscope (JEOL, Tokyo, Japan).

### Flow cytometry

Whole blood and mesenteric lymph nodes (MLNs) were collected from the rats. MLNs were mechanically mashed and filtered through a 100-mm nylon strainer to prepare a single-cell suspension. Mononuclear cells in blood were extracted using rat lymphocyte separation medium (Hao Yang Biological Technology, Tianjin, China). For the T helper 17 (Th17) assays, cells were stimulated with Cell Stimulation Cocktail (eBioscience, San Diego, CA, USA) for 6 h at 37 °C in a 5% CO2 environment and subsequently stained with CD4-phycoerythrin (PE) antibody. Fixation and permeabilization were performed using fix/perm buffer (Servicebio, Wuhan, China), followed by incubation with an IL-17-allophycocyanin (APC) antibody. For the Treg assay, cells were incubated with CD4-fluorescein isothiocyanate (FITC) antibody and CD25-PE antibody at 4 °C for 30 min in the dark. After fixation and permeabilization using reagents from Biolegend (San Diego, CA, USA), cells were stained with forkhead Box P3 (Foxp3)-Alexa Fluor 647 antibody (Biolegend) at 4 °C for 30 min in the dark. Cell analysis was performed using a flow cytometer (Beckman Coulter CytoFLEX, CA, USA), and data were analysed with FlowJo (Tree Star, Ashland, OR, USA).

### Real-time fluorescence quantitative PCR

Total RNA was extracted from the ileum and colon using TRIzol Reagent (Invitrogen Life Technologies, Waltham, USA) according to the manufacturer's instructions. Subsequently, cDNA was synthesized from the total RNA using the RevertAid First Strand cDNA Synthesis Kit (Thermo Scientific, Wilmington, USA). The qPCR primers were synthesized by Invitrogen Biotechnology (Carlsbad, CA, USA). GAPDH served as the internal reference gene. PCR was carried out on a real-time quantitative PCR system (Bio-Rad, California, USA) with an initial step at 95 °C for 3 min, followed by 40 cycles of 95 °C for 10 s and 55 °C for 30 s. Subsequently, a melting curve was generated (65 °C for 5 s and 95 °C for 20 s). Relative gene expression levels were determined using the 2^−ΔΔ^CT method. Primer sequences were as follows: GAPDH: 5’-GGG TGT GAA CCA CGA GAA AT-3’ and 5’-CCT TCC ACA ATG CCA AAG TT-3’; Foxp3: 5’-CAC CTT TCC AGA GTT CTT CCA CA-3’ and 5’-CGG ATG AGG GTG GCA TAG GT-3’; IL-17: 5’-GGA CTC TCC ACC GCA ATG AA-3’ and 5’-TTT CCC TCC GCA TTG ACA CA-3’. Each PCR was performed in technical triplicates.

### DNA extraction and 16S rRNA gene sequencing

Faecal samples from each group were collected aseptically and stored at − 80 °C for future analysis. Faecal DNA was extracted using the E.Z.N.A.® soil DNA Kit (Omega Biotek, Norcross, GA, USA) according to the manufacturer's instructions. The extracted DNA was assessed for quality on a 1% agarose gel, and DNA concentration and purity were determined using a NanoDrop 2000 UV‒vis spectrophotometer (Thermo Scientific). The V3-V4 region of the bacterial 16S rRNA gene was amplified using an ABI GeneAmp® 9700 PCR thermocycler (ABI, CA, USA) with the primer pair 338F (5’-ACT CCT ACG GGA GGC AGC AG-3’) and 806R (5’-GGA CTA CHV GGG TWT CTA AT-3’). PCR conditions included an initial denaturation at 95 °C for 3 min, followed by 27 cycles of denaturation at 95 °C for 30 s, annealing at 55 °C for 30 s, and extension at 72 °C for 45 s. The reaction concluded with a final extension step at 72 °C for 10 min. PCR amplicons were separated via 2% agarose gel electrophoresis and purified using an AxyPrep DNA Gel Extraction Kit (Axygen Biosciences, Union City, CA, USA). Quantification of amplicons was performed using QuantiFluor-ST (Madison, Promega, WI, USA). Subsequently, the amplicons were pooled in equimolar concentrations for paired-end sequencing on an Illumina MiSeq PE300 platform by Majorbio Bio-Pharm Technology Co. Ltd. (Shanghai, China) according to the manufacturer’s instructions.

### Microbiota data analysis

After demultiplexing, sequences underwent quality filtering with fastp (0.19.6)^32^ followed by merging using FLASH (v1.2.11). High-quality sequences were denoised with DADA2^33^ in the QIIME2^34^ (version 2020.2) pipeline with recommended parameters, leading to the generation of amplicon sequence variants (ASVs). To account for sequencing depth variability, rarefaction was applied, with all samples standardized to 20,000 sequences while maintaining an average Good's coverage of 97.90%. Taxonomy assignment was performed by the naive Bayes consensus taxonomy classifier within QIIME2 and the SILVA 16S rRNA database (version 138). Bioinformatic analysis was conducted using the Majorbio Cloud platform (https://cloud.majorbio.com). Alpha diversity indices, including observed ASVs and the Shannon index, were computed using Mothur (version 1.30.1)^35^. Microbial community similarities were assessed using principal coordinate analysis (PCoA) based on Bray‒Curtis dissimilarity via the Vegan v2.5-3 package. The PERMANOVA test, also in Vegan v2.5-3, was used to determine treatment-related variation and significance. To identify significantly abundant taxa (phylum to genera) among groups (LDA score > 3, *P* < 0.05), we performed linear discriminant analysis effect size (LEfSe)^36^ (http://huttenhower.sph.harvard.edu/LEfSe).

### Ethical approval and consent to participate

The study was approved by the Ethics Committee of Sichuan University West China Medical for Animal Experimentation (K2019002-2).

## Results

### ATRA alleviated arthritis in CIA rats

**Figure 2:**
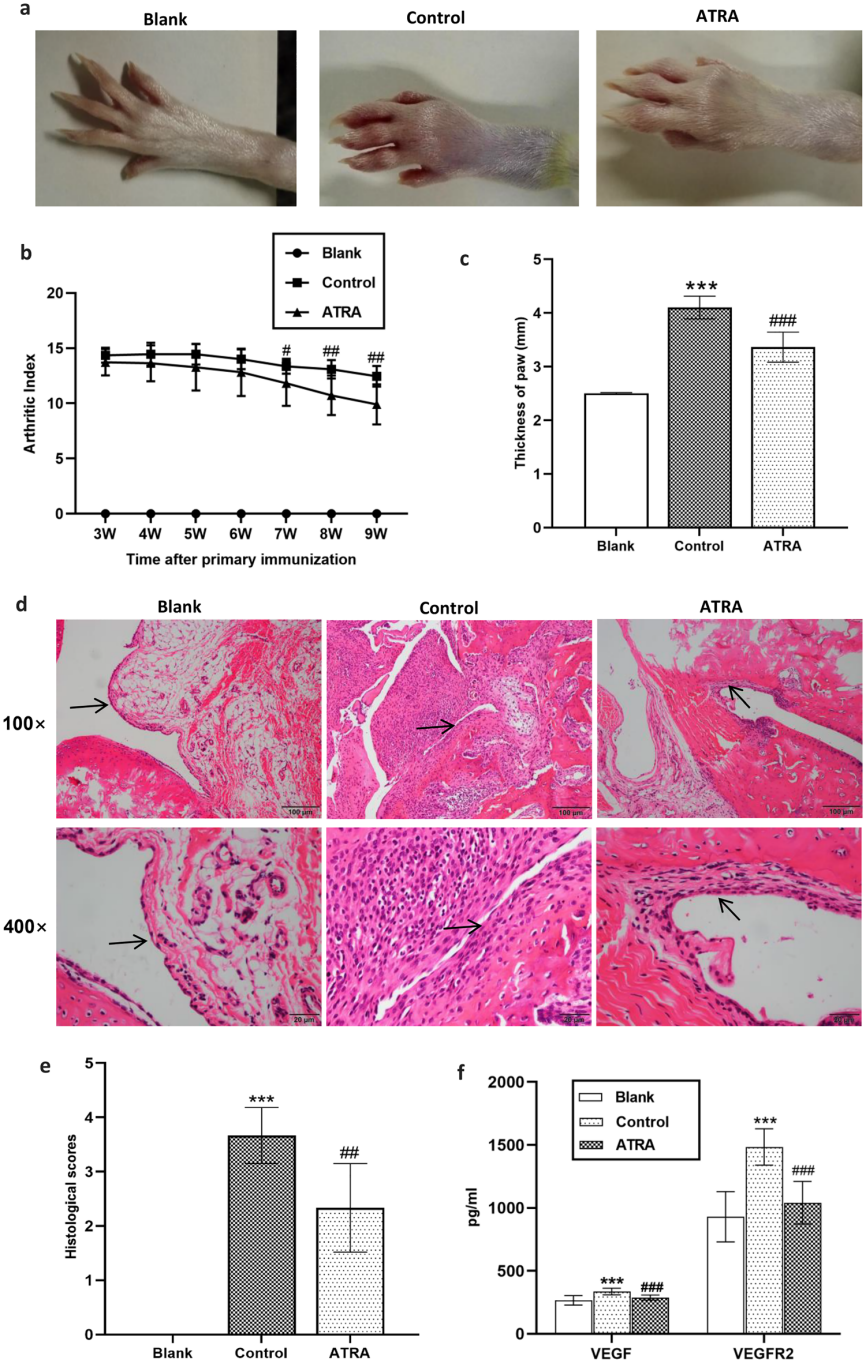
Antiarthritic effects of ATRA in CIA rats. (**a**) Representative images from the macroscopic observation of hind legs. (**b**) The arthritis index was measured weekly beginning 3 weeks after the primary immunization (N ≥ 10 per group). (**c**) The thickness of paws was assessed 9 weeks after the primary immunization (N ≥ 10 per group). (**d**) Representative images of H&E staining of ankle joints. (**e**) Histopathology assessments of ankle joints were scored to assess the severity of arthritis (N = 6 per group). (**f**) Rat serum concentrations of VEGF and VEGFR2 were quantified via ELISA (N ≥ 10 per group). Bars, SD; ^***^P < 0.001 versus Blank; ^#^*P* < 0. 05, ^##^*P* < 0. 01, ^###^*P* < 0.001 versus Control. → Synovium.

Following immunization with bovine type II collagen, rats exhibited arthritis-associated symptoms, including limps, paw and joint swelling, weight loss, and a substantial increase in the arthritis index (AI) (Fig. 2a–c). In addition, histological analyses revealed synovial inflammation, pannus formation, cartilage erosion, and subchondral bone remodelling in the ankle joints of CIA rats (Fig. 2d–e). After treatment with ATRA, paw and joint swelling was relieved, and the AI score was markedly reduced starting 7 weeks after primary immunization (Fig. 2a–c, e). Moreover, histological analysis showed that ATRA attenuated synovial inflammation and reduced articular cartilage and bone damage, and the histological score decreased significantly (*P* = 0.002, Fig. 2d–e). We also measured the serum angiogenesis-related factors VEGF and VEGFR2. ATRA led to a significant reduction in both VEGF and VEGFR2 levels (*P* < 0.001), as depicted in Fig. 2f. There were no obvious changes in weight, organ coefficients or H&E staining of the liver, kidney, spleen and thymus in CIA rats after ATRA treatment (Supplementary Fig. S1).

### ATRA regulated the systemic and intestinal immune response

**Figure 3:**
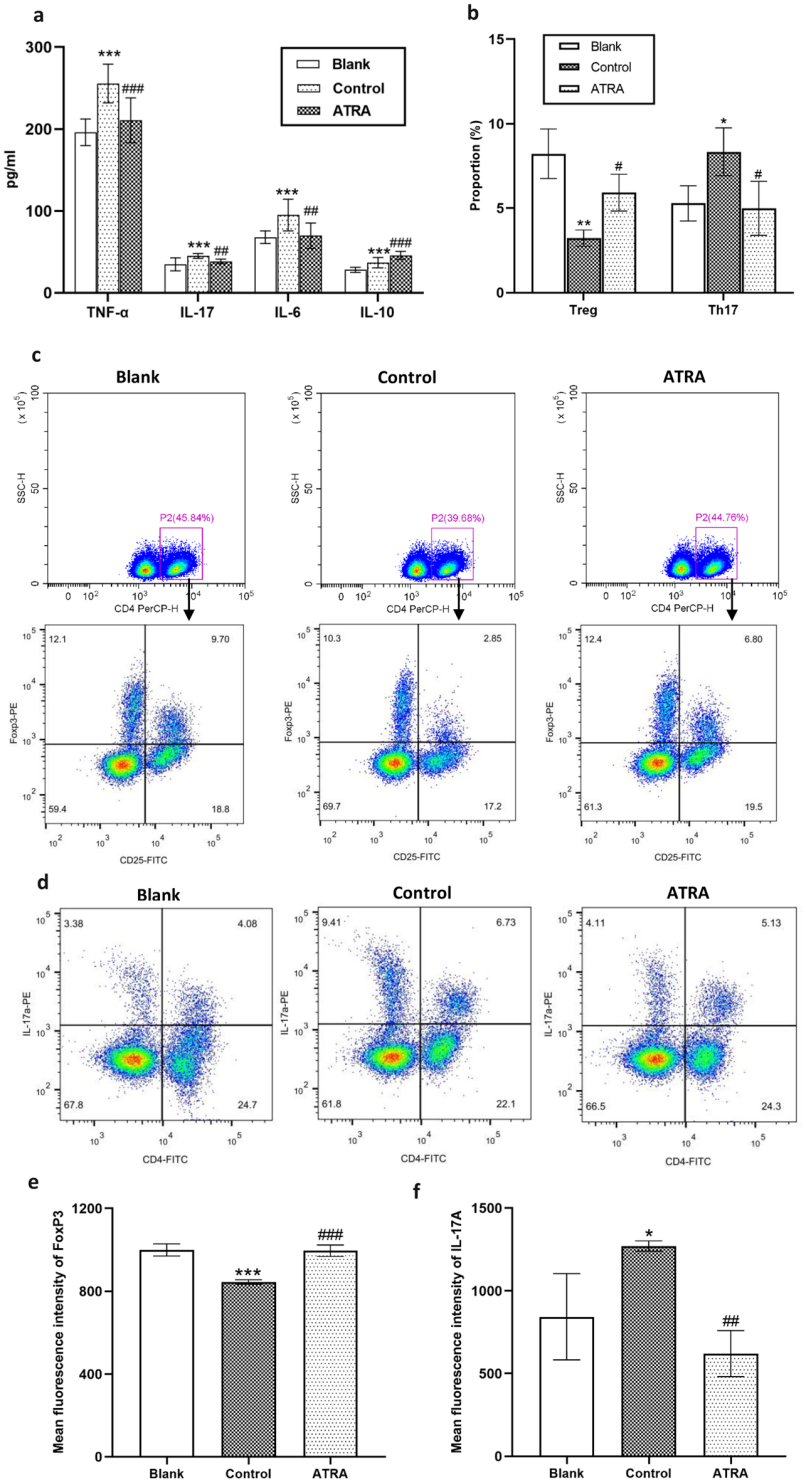
Effects of ATRA on systemic inflammatory responses in CIA rats. (**a**) Rat serum concentrations of IL-6, IL-17, TNF-α and IL-10 were quantified via ELISA (N ≥ 10 per group). (**b**) The proportions of CD4^+^CD25^+^FoxP3^+^ Tregs and CD4^+^IL-17A^+^ Th17 cells detected by flow cytometric analysis are represented as the mean ± SD (N = 3 per group). Representative images of flow cytometric analysis of the CD4^+^CD25^+^FoxP3^+^ Treg cell (**c**) and CD4^+^IL-17A^+^ Th17 cell (**d**) proportions in the peripheral blood mononuclear cells (PBMCs) of rats. Mean fluorescence intensity of FoxP3 (**e**) and IL-17A (**f**) at single cell levels. Bars, SD; ^*^P < 0. 05, ^**^P < 0. 01; ^***^P < 0.001 versus Blank; ^#^*P* < 0. 05, ^##^*P* < 0. 01; ^###^*P* < 0.001 versus Control.

Serum cytokine levels were assessed by ELISA to evaluate the systemic immune response to inflammation. ATRA considerably reduced the levels of the proinflammatory cytokines TNF-α, IL-6 and IL-17 while increasing the levels of the anti-inflammatory cytokine IL-10, as shown in Fig. 3a. Flow cytometry was utilized to assess Th17 and Treg cell proportions in the peripheral blood mononuclear cells (PBMCs) of rats. In CIA rats, the Treg frequency in CD4^+^ T cells decreased, while the Th17 frequency increased significantly compared to that in rats in the blank group (*P* < 0.001). However, ATRA treatment effectively reversed the Th17/Treg imbalance in PBMCs (Fig. 3b–d). We further examined the mean fluorescence intensity (MFI) of foxp3 and IL-17A at the single-cell level, as shown in Fig. 3e–f. ATRA significantly increased the MFI of FoxP3 and decreased the MFI of IL-17A (*P* < 0.001 and *P* = 0.008 respectively).

**Figure 4:**
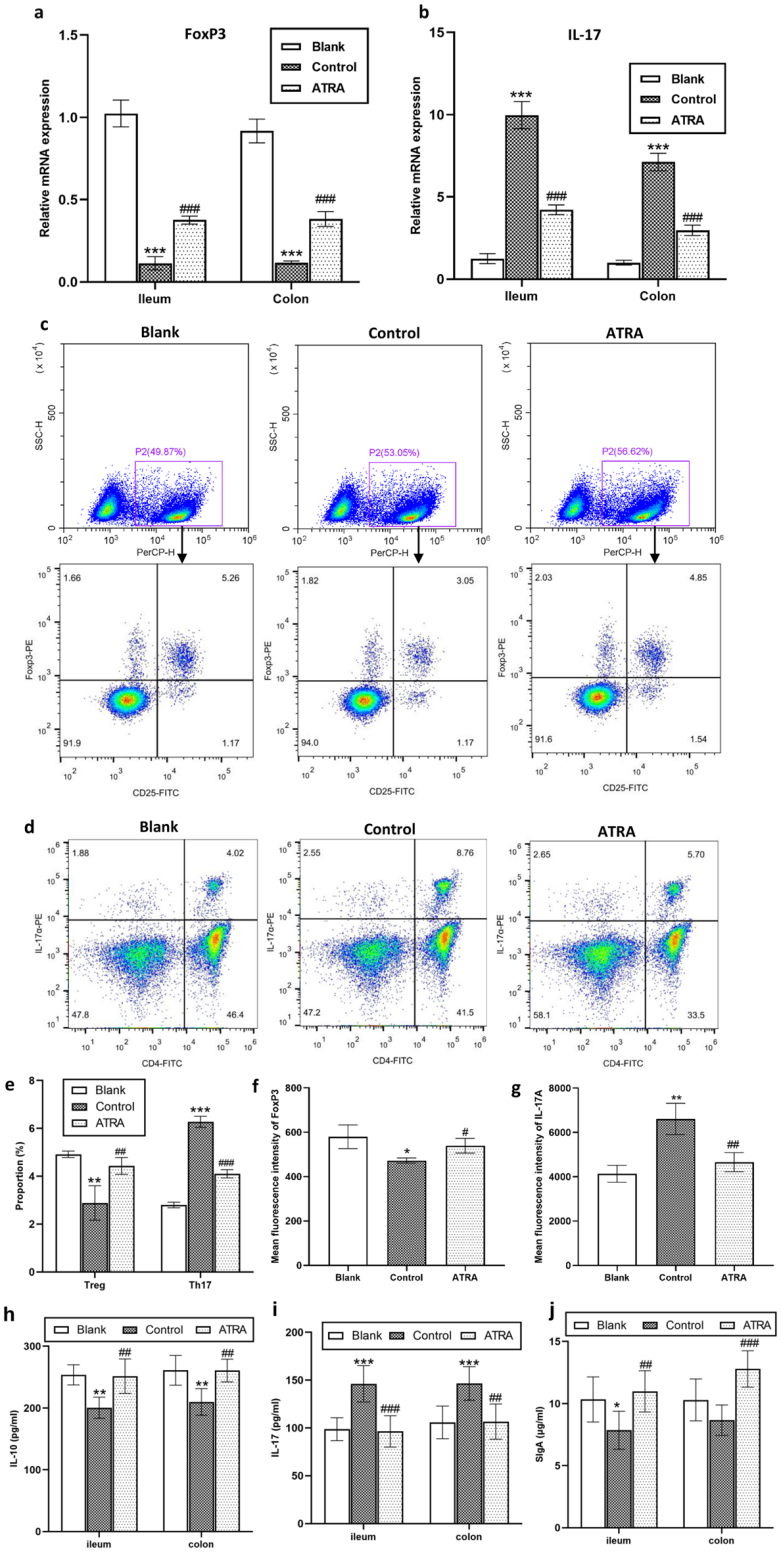
Modulation of intestinal immunity in CIA rats by ATRA. Relative expression levels of FoxP3 mRNA (**a**) and IL-17 mRNA (**b**) in rat colon and ileum tissues were determined by RT‒qPCR (N = 6 per group). Representative images of flow cytometric analysis of the CD4^+^CD25^+^FoxP3^+^ Treg cell (**c**) and CD4^+^IL-17A^+^ Th17 cell (**d**) proportions in mesenteric lymph nodes (MLNs). (**e**) The proportions of CD4^+^CD25^+^FoxP3^+^ Tregs and CD4^+^IL-17A^+^ Th17 cells are represented as the mean ± SD (N = 3 per group). Mean fluorescence intensity of FoxP3 (**f**) and IL-17A (**g**) at single cell levels. The levels of IL-10 (**h**), IL-17 (**i**) and SIgA (**j**) secretion in the rat ileum and colon were quantified via ELISA (N = 6 per group). Bars, SD; ^*^P < 0. 05, ^**^P < 0. 01, ^***^P < 0.001 versus Blank; ^#^*P* < 0. 05, ^##^*P* < 0. 01, ^###^*P* < 0. 001 versus Control.

Immune responses outside the intestines can be influenced by immune cell proliferation and differentiation within the intestines^11^. Therefore, we assessed the relative mRNA expression of FoxP3 and IL-17 in the colon and ileum. As illustrated in Fig. 4a, b, CIA rats exhibited significantly decreased mRNA expression levels of FoxP3 and increased expression levels of IL-17 in the colon and ileum compared to the blank group (*P* < 0.001). However, the changes in these levels were reversed after ATRA intervention. Furthermore, we investigated the proportions of CD4^+^CD25^+^FoxP3^+^ Treg cells and CD4^+^IL-17A^+^ cells in MLNs using flow cytometry. Treg frequency decreased, while Th17 frequency increased in CIA rats when compared to the blank group. ATRA treatment remarkably decreased the Th17/Treg ratio (Fig. 4c–e). We further examined the MFI of foxp3 and IL-17A, as shown in Fig. 4f–g. ATRA significantly increased the MFI of FoxP3 and decreased the MFI of IL-17A (*P* = 0.030 and *P* = 0.009 respectively). Moreover, ELISA analysis showed substantial increases in the secretion of IL-10 and SIgA, along with decreases in IL-17 following ATRA treatment (Fig. 4h–j).

### ATRA improved intestinal barrier function in CIA rats.

**Figure 5:**
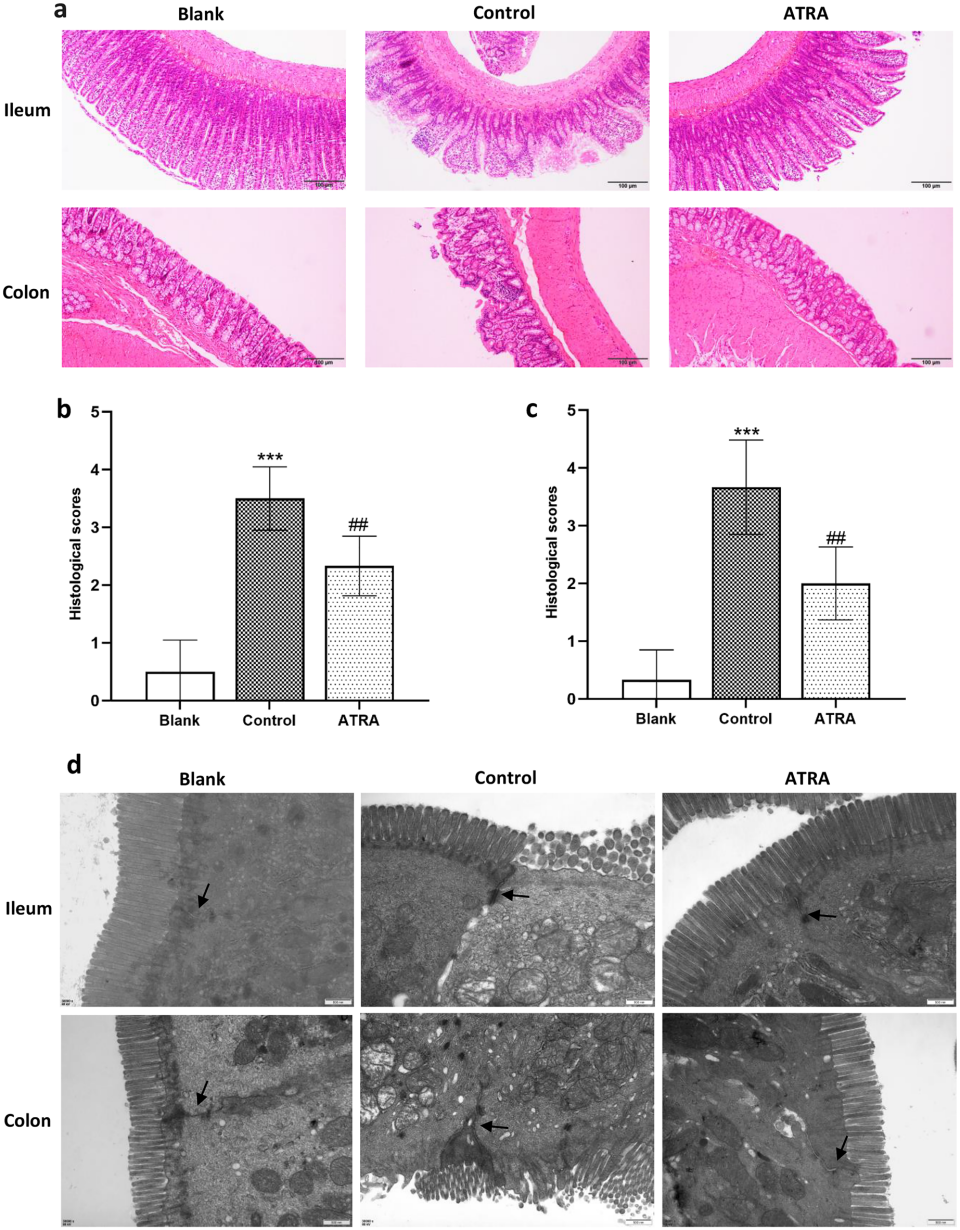
Effects of ATRA on gut morphology and ultrastructure in CIA rats. (**a**) Representative images of the rat colon and ileum stained with H&E. Pathological sections of rat ileum (**b**) and colon (**c**) were scored and are represented as the mean ± SD (N = 6 per group). **(d)** Transmission electron microscopy (TEM) images of the rat colon and ileum (12,000 × ; N = 3 per group). → Tight junction. ^***^P < 0.001 versus Blank; ^##^*P* < 0. 01 versus Control.

We first utilized H&E staining to identify pathological changes in the colon and ileum tissues. As shown in Fig. 5a, CIA rats exhibited signs of intestinal inflammation, including lymphocyte infiltration within the rat intestinal mucosal layer, accompanied by localized epithelial damage, villous atrophy, and structural disarray. Subsequent to ATRA intervention, a notable restoration of rat intestinal inflammation occurred, and the histological score decreased significantly (*P* = 0.005 for ileum and *P* = 0.002 for colon, Fig. 5b, c). To further explore intestinal epithelial TJs and ultrastructural characteristics, we employed TEM. As shown in Fig. 5d, the control group exhibited damage to TJs. In severe cases, there was cell necrosis, villous atrophy or dissolution, accompanied by blurring or the dissolution of TJs. Following ATRA intervention, we observed a partial recovery of TJs.

**Figure 6:**
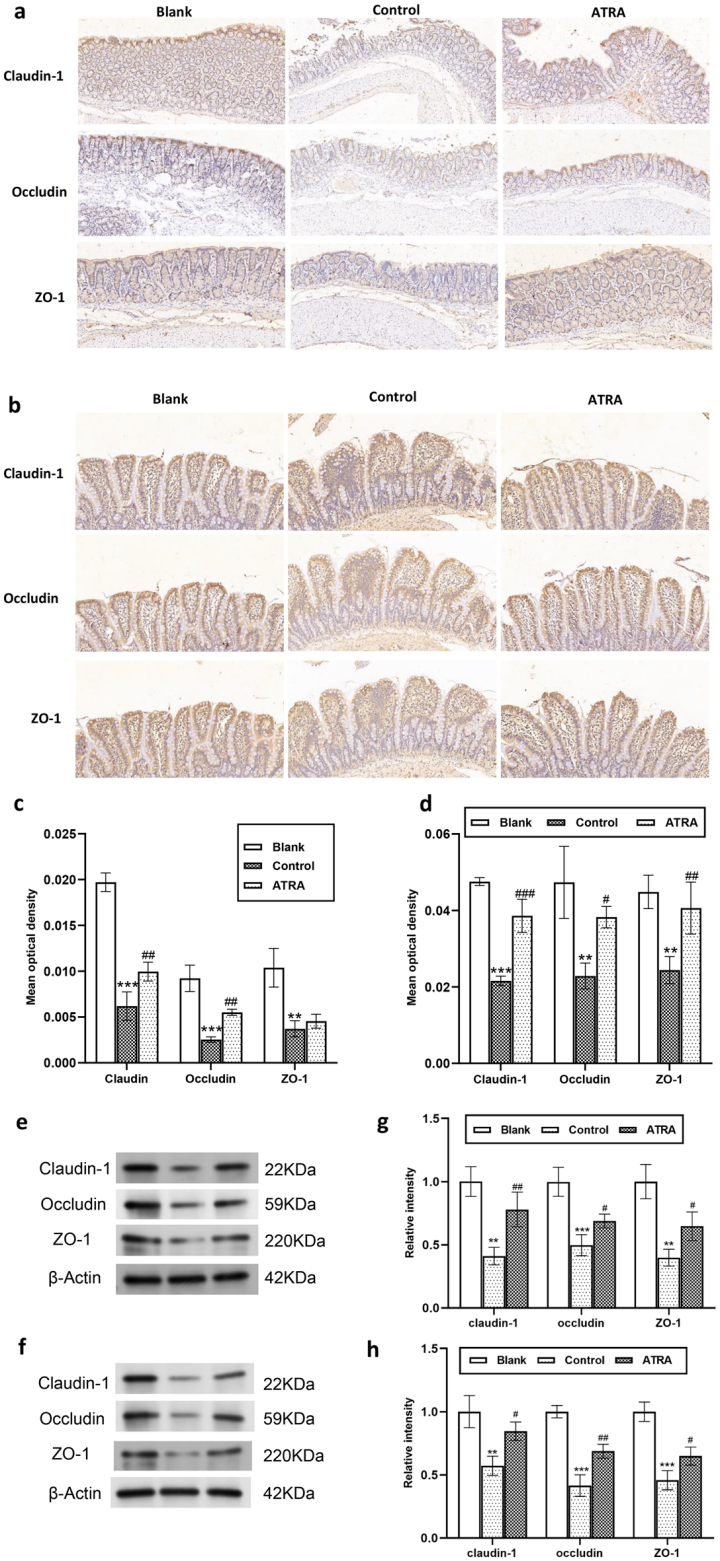
Expression of tight junction proteins in the rat colon and ileum. Immunohistochemical staining of the claudin-1, occludin, and ZO-1 proteins in the rat colon (**a**) and ileum (**b**). Quantitative analysis of claudin-1, occludin, and ZO-1 protein expression in the rat colon (**c**) and ileum (**d**) (N = 3 per group). The protein expression of claudin-1, occludin, and ZO-1 in the rat colon (**e**) and ileum (**f**) was calculated by western blotting. Depicted western blots are representatives from three independent experiments. Quantification of the relative intensity of the western blot images of colon (**g**) and ileum (**h**) were represented as the mean ± SD. Bars, SD; ^**^P ≤ 0.01, ^***^P < 0.001 versus Blank; ^#^*P* < 0. 05, ^##^*P* < 0. 01; ^###^*P* < 0.001 versus Control.

Then, we performed IHC and western blotting to measure the expression of TJ proteins, including Claudin-1, Occludin and ZO-1. As shown in Fig. 6, the control group exhibited the lowest levels of all three proteinxs. After ATRA treatment, there was a notable increase in the expression of these proteins (except for colonic ZO-1 based on IHC). These results collectively suggest that ATRA may promote the restoration of colonic and ileal epithelial barriers in CIA rats.

### ATRA modulated the composition of the intestinal microflora of CIA rats

To determine the influence of ATRA on the structure and composition of the intestinal flora of CIA rats, we collected faeces and performed 16S rRNA sequencing to evaluate the structure of the gut flora. As shown in Supplementary Fig. S2a, b, the control group had the largest mean Shannon index, and ATRA significantly reduced the Shannon index (*P* = 0.017), suggesting that ATRA may modify the excessively diverse intestinal flora of CIA rats by shifting its composition in an anti-arthritic direction.

**Figure 7:**
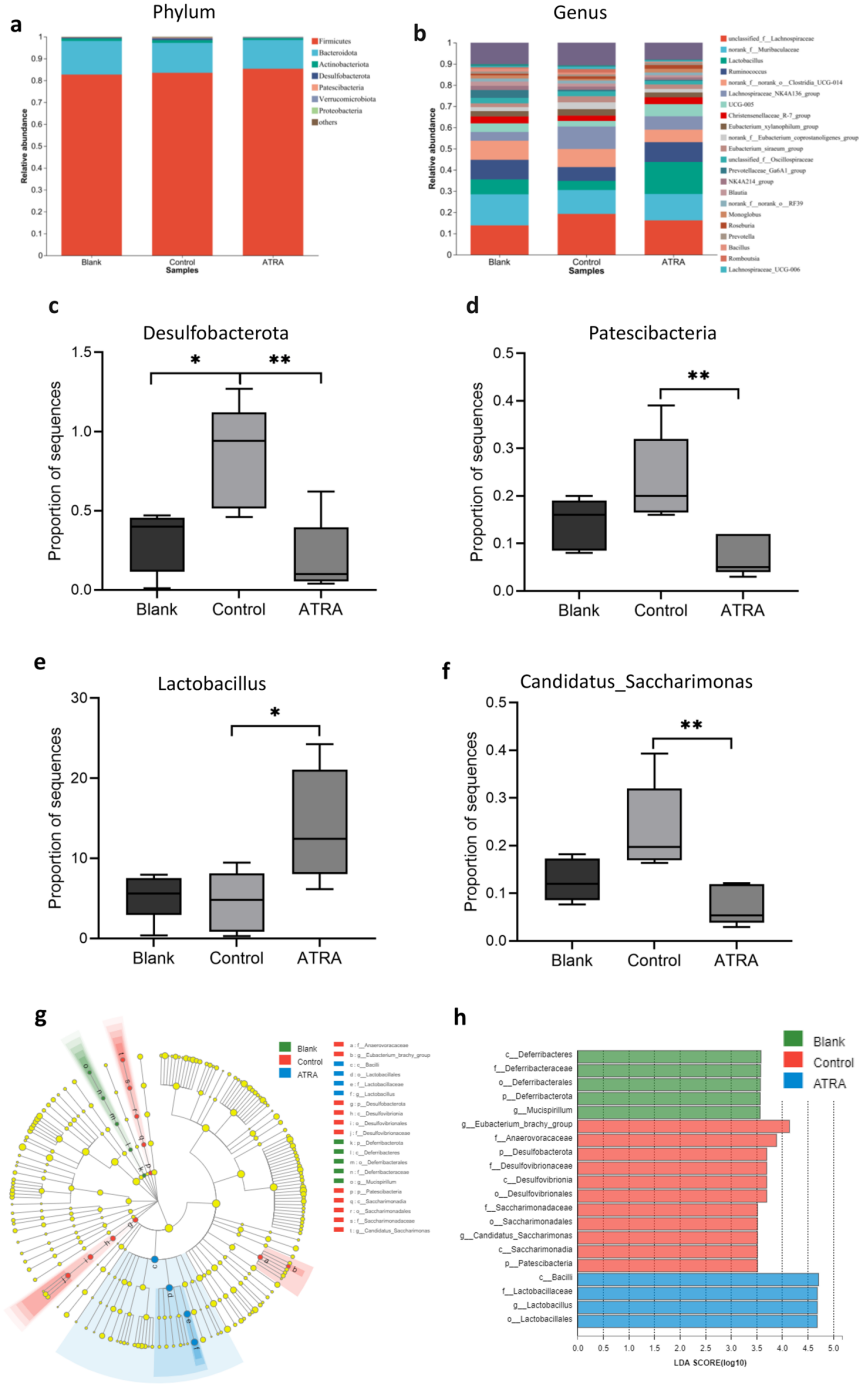
Effects of ATRA on the bacterial composition in the gut of CIA rats. Bacterial composition at the phylum (**a**) and genus (**b**) levels. Quantitative analyses of the relative abundances of *Desulfobacterota *(**c**), *Patescibacteria* (**d**), *Lactobacillus* (**e**) and *Candidatus_Saccharimonas* (**f**) among the different groups. (**g**) Cladogram produced by linear discriminant analysis effect size (LEfSe) analysis. (**h**) Histogram of the linear discriminant analysis (LDA) scores for differentially abundant properties. LDA scores ≥ 3.0 are shown. Statistical significance was determined using the Kruskal‒Wallis rank sum test, followed by Tukey's HSD test. N = 5 per group; Bars, SD; ^*^P ≤ 0.05, ^**^P ≤ 0.01.

The Venn diagram in Supplementary Fig. S2c illustrates the overlap and unique ASVs among the three groups. Moreover, principal coordinates analysis (PCoA) revealed a modest difference in gut microbiota structure among the three groups (R = 0.172, *P* = 0.039, Supplementary Fig. S2d). In addition, we analysed the gut microbiota composition at both the phylum and genus levels (Fig. 7a, b, Supplementary Fig. S2e, f). As illustrated in Fig. 7a, the predominant phyla identified were Firmicutes, Bacteroidota, Actinobacteria, Desulfobacterota, and Patescibacteria. Notably, there was an increase in the abundance of Desulfobacterota and Patescibacteria in the CIA rats compared to the blank group, and these alterations were reversed following ATRA treatment (*P* < 0.05; Fig. 7c, d). At the genus level, there was a significant increase in the abundance of *Lactobacillus* (*P* = 0.024), while the abundance of *Candidatus_Saccharimonas* significantly decreased (*P* = 0.005; Fig. 7e, f).

The cladogram highlighted the enriched species that are crucial for distinguishing the three groups. As depicted in Fig. 7g, h, the blank group exhibited significant enrichment of 4 taxa, predominantly *Deferribacteraceae* and *Mucispirillum*. The control group featured 11 biomarkers, primarily *Eubacterium_brachy_group*, *Anaerovoracaceae* and *Desulfovibrionaceae*. Furthermore, the ATRA group has 4 distinctive taxa, with *Lactobacillus* dominating in terms of composition.

## Discussion

A growing body of research has highlighted the interplay between the gut microbiota, intestinal permeability and immune responses, which can impact joint inflammation^11,37,38^. ATRA, with its potential to regulate inflammatory factors and immune cell differentiation, has emerged as a promising candidate for mitigating RA. However, whether the gut-joint axis plays a role in this process is unclear. Our study provides evidence that ATRA can reverse the imbalance in Th17/Treg cells in the intestine, elevate the levels of intestinal IL-10 and SIgA, decrease the intestinal IL-17 level, restore intestinal TJs, and modulate the composition of the gut microbiota in CIA rats. Meanwhile, the symptoms of arthritis in CIA rats were significantly alleviated. These findings suggest that ATRA alleviation of RA might be associated with the modulation of gut microbiota and intestinal immunity.

In the early 1990s, physicians had already detected microscopic gut inflammation in patients with arthritis, which led to the discovery of the well-known link between gastrointestinal inflammation and spondyloarthritis (SpA)^39–41^. In SpA, subclinical gut inflammation occurs in more than 50% of patients^42–44^. Gut inflammation, characterized by the expansion of abnormally activated innate immune cells, may lead to disruption of the intestinal barrier, resulting in increased permeability and bacterial translocation. These processes can trigger inflammation at extraintestinal sites and contribute to autoimmunity, particularly in individuals with genetic susceptibility^45–47^. In our study, we observed varying degrees of intestinal inflammation in CIA rats, along with impaired intestinal mucosal barrier function. Notably, ATRA demonstrated significant efficacy in ameliorating these conditions.

Th17 cells, which are a subset of T lymphocytes, primarily secrete the proinflammatory cytokine IL-17. Treg cells are characterized by the expression of FoxP3 and secretion of anti-inflammatory cytokines such as IL-10 and TGF-β^48^. The balance of Th17/Treg plays a pivotal role in preventing autoimmune reactions and maintaining immune homeostasis^49^. In this study, we observed that ATRA decreased the proportion of Th17 cells and increased the proportion of Treg cells in both MLNs and PBMCs. Additionally, ATRA reduced the levels of proinflammatory cytokines while increasing the concentrations of anti-inflammatory cytokines in the peripheral blood and intestine. Previous studies have suggested that when the integrity of the intestinal mucosal barrier is damaged, immune cells may infiltrate the joints through the bloodstream^14,50^. In our study, we observed consistent changes in Th17 and Treg cells, as well as inflammatory factors, in both the intestine and blood. Based on these findings, we propose that the improvement in the Treg/Th17 balance and the reduction in inflammatory factors in peripheral blood may be associated with ATRA-mediated modulation of intestinal immunity.

In recent years, numerous studies have demonstrated an altered gut microbiota in RA patients, suggesting that mucosa-microbe interactions play a significant role in the development of RA^7,10,51^. In the present study, ATRA significantly altered the excessively diverse intestinal flora of CIA rats and altered the gut microbiota composition, as characterized by a reduced abundance of Desulfobacterota and an increased abundance of *Lactobacillus*.

Desulfobacterota typically possess the ability to reduce sulfate as part of their metabolism, often resulting in the production of hydrogen sulfide. Previous studies have established the associations between *Desulfovibrio* and various diseases, including intestinal inflammation, Parkinson's disease, joint inflammation and diabetes^52^. The mechanism by which *Desulfovibrio* spp. alleviate inflammation is associated with the regulation of immune cells. Studies have indicated an increased level of CD11b^+^, B cells, CD8^+^ T cells and Treg cells in the MLNs of mice enriched with sulfate-reducing bacteria (SRB)^53^. Enrichment of SRB has also been shown to induce Th17 and Treg immune responses in the MLNs of germ-free mice^53^. Additionally, oral administration of *Desulfovibrio desulfuricans* exacerbated atherosclerotic lesions in Apoe−/−mice, thereby increasing intestinal permeability and systemic inflammation^54^. Moreover, Singh et al.^55^ found that *Desulfovibrio vulgaris* increased intestinal barrier permeability by upregulating the expression of the transcription factor Snail1 and disrupting occludin localization.

**Figure 8:**
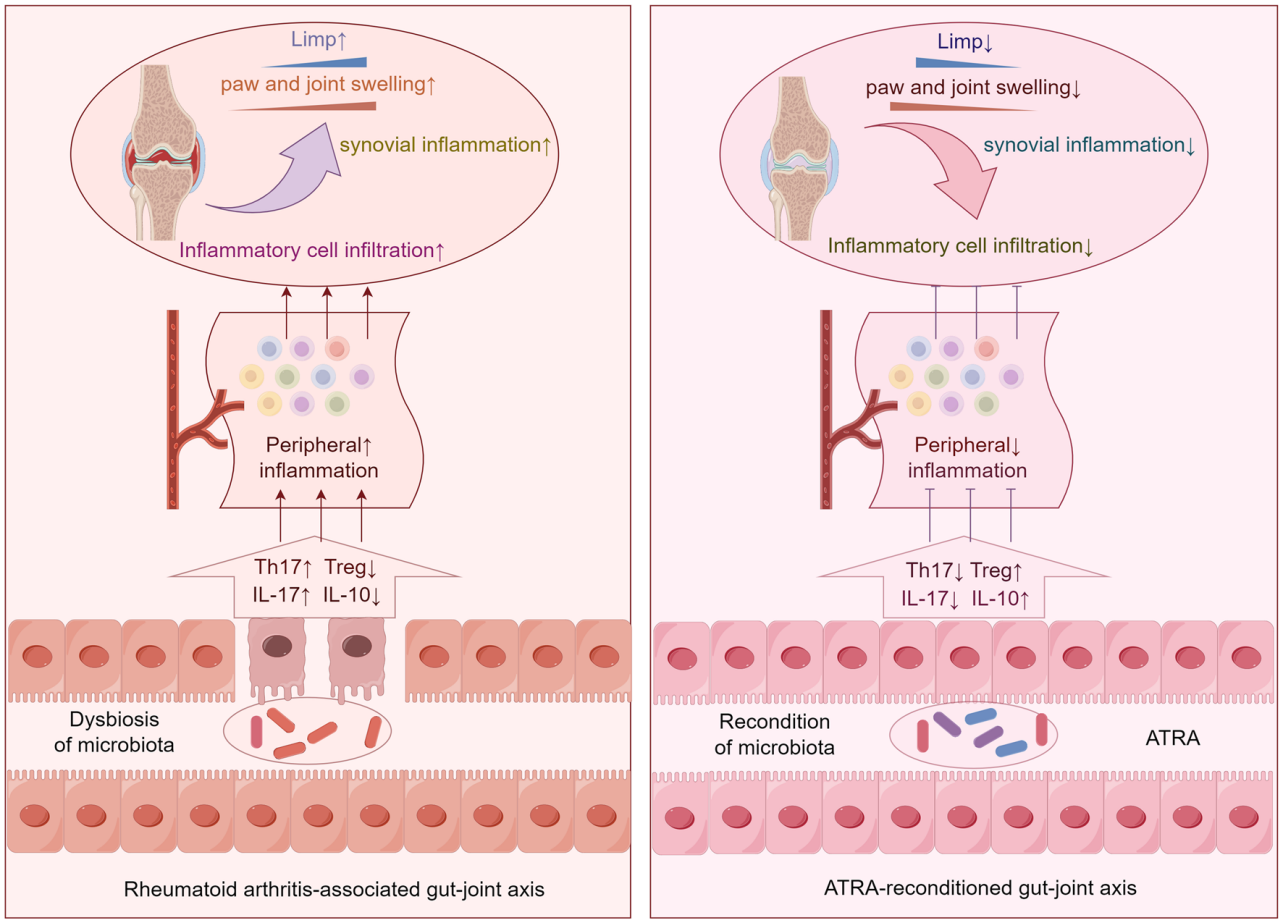
Schematic diagram of our hypotheses regarding the mechanisms through which ATRA alleviates RA (by Figdraw). By modulating the gut microbiota composition, ATRA inhibits Th17 cell differentiation while promoting Treg cell differentiation, thereby regulating the production of inflammatory factors. Consequently, this regulation alleviates intestinal inflammation, decreases intestinal mucosal permeability, and further ameliorates systemic inflammatory responses, ultimately resulting in the alleviation of arthritis.

*Lactobacillus* bacteria are known for their beneficial properties. Previous studies have shown that *Lactobacillus* species can reinforce the gut barrier and regulate T-cell differentiation. This helps maintain immune tolerance and suppress excessive immune responses^56,57^. Numerous studies have demonstrated the significant improvement in RA patients mediated by *Lactobacillus* spp. Fan et al.^58^ found that *Lactobacillus casei* CCFM1074 improved the condition of CIA by balancing Treg/Th17 populations, modulating the gut microbiota, and regulating plasma metabolites. Additionally, *L. rhamnosus* CNCM-I upregulated the expression of zonula occludens (ZO)-1, occludin, and claudin proteins in Caco-2 cells^59^. These findings are in line with the results of our study. The mechanisms by which *Lactobacillus* spp. improve arthritis may be related to the promotion of short-chain fatty acid (SCFA) production. These compounds regulate various innate immune activities and modulate external inflammatory responses and oxidative stress levels^60,61^. For instance, butyrate can inhibit the proliferation of antigen-specific B cells and plasma cells, as well as the cytokine production of natural killer T cells (NKT cells), which are closely associated with arthritis and tissue degeneration^62^. Consequently, we propose that ATRA can impact intestinal immunity by altering the gut microbiota composition. Figure 8 illustrates our hypotheses regarding the mechanisms through which ATRA alleviates RA.

Several limitations of this study should be noted. First, while our findings are promising, translating results from animal models to human patients may not necessarily yield identical outcomes. Second, while we observed changes in the gut microbiota, intestinal permeability and immunity, the precise molecular mechanisms underlying these effects were not fully elucidated in this study, indicating the need for further mechanistic explorations. Third, in the current study, we did not conclusively establish whether ATRA alleviates RA by modulating the gut microbiota. Subsequent investigations that involve removing the intestinal flora will further elucidate the causal relationship.

## Conclusions

Based on a CIA model, this study shows that ATRA can alleviate the symptoms of arthritis, improve synovial hyperplasia, reduce serum proinflammatory cytokine levels, and reduce the Th17/Treg ratio. Furthermore, ATRA-mediated alleviation of arthritis might be associated with modulating the gut microbiota, balancing Th17/Treg cells, reducing the inflammatory reaction in the ileum and colon and protecting the intestinal barrier. Our findings provide novel insights into ATRA-mediated alleviation of arthritis and the potential role of ATRA as a modulator of the gut microbiota in treating RA.

### Supplementary Information

Supplementary Figures.

## Data Availability

The raw data of 16S rRNA amplicon sequences were deposited in Sequence Read Archive of NCBI (accession code SRP463891). The other data presented in this study are openly available in FigShare at 10.6084/m9.figshare.24637473.

## Abbreviations

RA: Rheumatoid arthritis
ATRA: All-trans retinoic acid
CIA: Collagen-induced arthritis
DMARD: Disease-modifying antirheumatic drug
ELISA: Enzyme-linked immunosorbent assay
IFA: Incomplete Freund's adjuvant
AI: Arthritis index
H&E: Haematoxylin and eosin
TJ: Tight junction
IHC: Immunohistochemistry
TEM: Transmission electron microscopy
MLN: Mesenteric lymph nodes
VEGF: Vascular endothelial growth factor
VEGFR2: Vascular endothelial growth factor receptor 2
Treg: Regulatory T cells
Th17: T helper 17
PBMC: Peripheral blood mononuclear cells
ASVs: Amplicon sequence variants
PCoA: Principal coordinate analysis
FOXP3: Forkhead box protein 3
mAb: Monoclonal antibody
SIgA: Secretory immunoglobulin A
TNF-α: Tumor necrosis factor-α
IL: Interleukin
LEfSe: Analysis effect size
SpA: Spondyloarthritis
LDA: Linear discriminant analysis
