# Supplementary material for: All-trans retinoic acid alleviates collagen-induced arthritis and promotes intestinal homeostasis
Source: Sci Rep. 2024 Jan 20;14:1811. doi: 10.1038/s41598-024-52322-x (PMC10799902; doi:10.1038/s41598-024-52322-x)
Supplement: Supplementary file 1 — Supplementary Figures. [file 41598_2024_52322_MOESM1_ESM.pdf]

All-trans retinoic acid alleviates collagen-induced arthritis and promotes intestinal homeostasis

Yiqi Zhang[1], Yating Luo[1], Jiangchun Shi[1], Yumeng Xie[1], Huangfang Shao[1], Yun Li[1,2*]

[1]Department of Nutrition and Food Hygiene, West China School of Public Health and West China Fourth Hospital, Sichuan University, Chengdu, Sichuan, China;

[2]Healthy Food Evaluation Research Center, Sichuan University, Chengdu, China.

* Corresponding author: Yun Li, e-mail: liyun_610@163.com

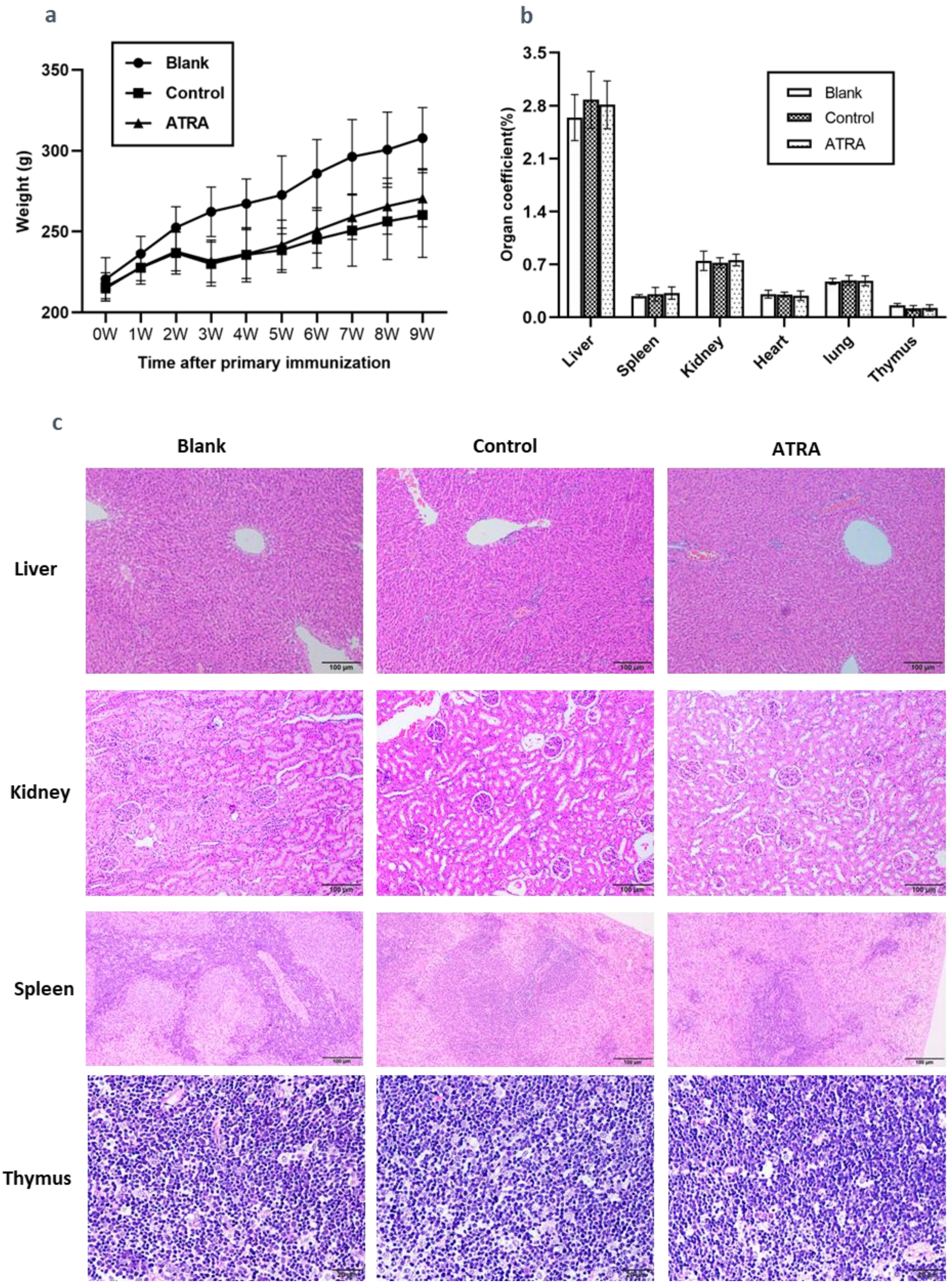

Supplementary Figure S1: ATRA has no effect on collagen-induced arthritis (CIA) rats weight, organ coefficients and tissue histopathology. **(a)** Body weight was recorded weekly. **(b)** Organ coefficients were calculated as organ weight(g)/body weight(g)×100. **(c)** Representative histopathological images of liver, kidney, spleen and thymus. Bars, SD; N≥10 per group.

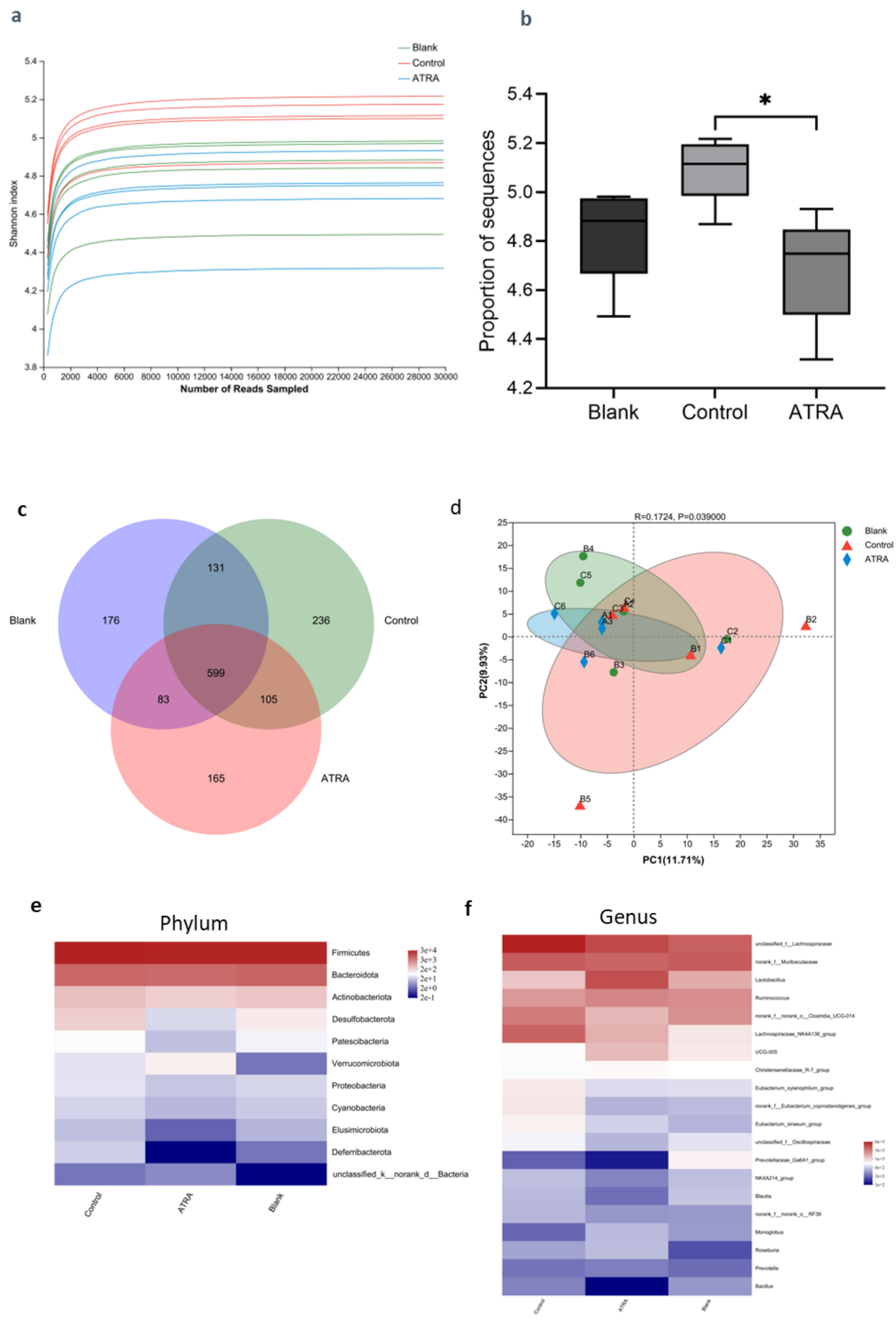

Supplementary Figure S2: Gut Microbiota Analysis in Rats. **(a)** Dilution curve presenting the relationship between sequencing depth and Shannon diversity index. **(b)** A box diagram of the Shannon index differences between the groups. **(c)** Venn diagram at the ASV level. **(d)** PCoA plots assessed by PERMANOVA among the three groups. **(e)** Heat map presenting relative abundance of microbial phyla. **(f)** Heat map of the top 20 microbial genera relative abundance. Heat maps were generated using the "pheatmap" packages of R software (version 3.3.1) (https://www.r-project.org/). N=5 per group.

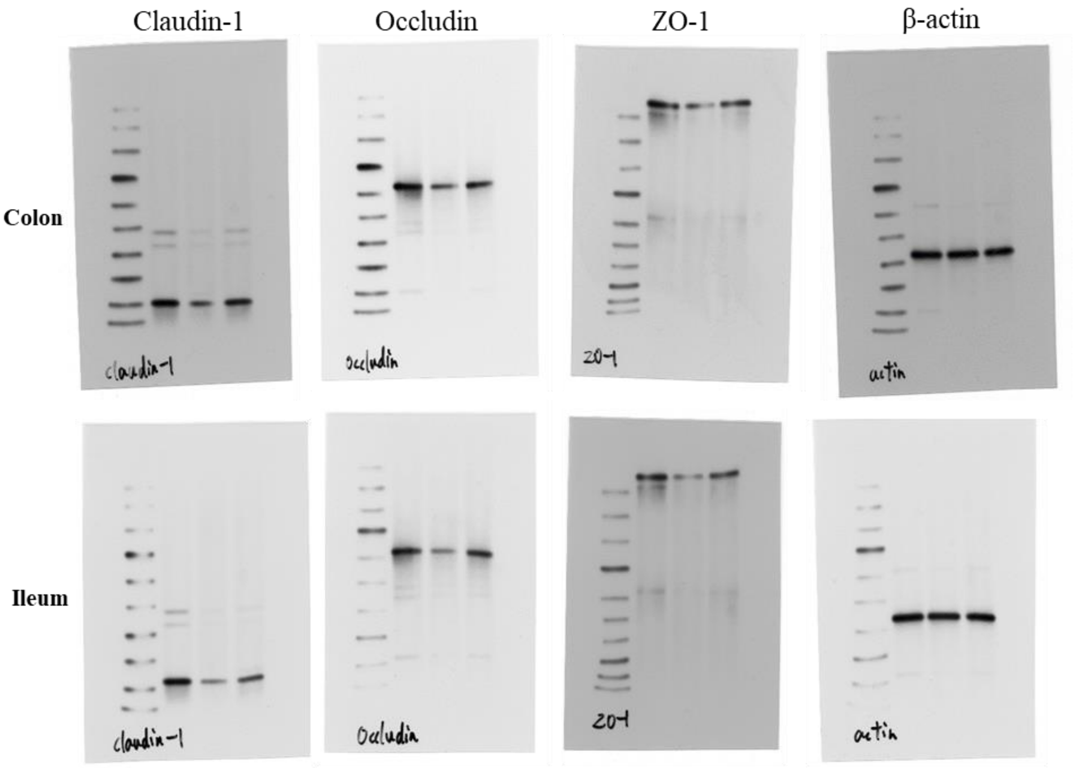

Supplementary Figure S3: Uncropped original images of Western blots in Fig. 6.
